# Redesign of Bedside Supply Carts to Improve Emergency Department Workflows: Mixed Methods Participatory Design

**DOI:** 10.2196/80861

**Published:** 2026-01-28

**Authors:** Kat Hefter, Sammy Dreibelbis, Theresa Haupt, Amelia McIver, Agnes Wang, Julia Dwight, Ogechi Nwodim, Neil Ray

**Affiliations:** 1 Department of Bioengineering, School of Engineering and Applied Sciences, University of Pennsylvania, 210 S. 33rd St., Suite 240, Skirkanich Hall, Philadelphia, PA, 19104, United States; 2 Department of Integrated Product Design, School of Engineering and Applied Sciences, University of Pennsylvania, Philadelphia, PA, United States; 3 Department of Data Sciences, School of Engineering and Applied Sciences, University of Pennsylvania, Philadelphia, PA, United States; 4 Emergency Department, University of Pennsylvania Health System, Philadelphia, PA, United States; 5 Department of Emergency Medicine, Perelman School of Medicine, University of Pennsylvania, Philadelphia, PA, United States

**Keywords:** cluster analysis, emergency department, ergonomic workflow, human-centered design, inventory, medical supply cart, patient care

## Abstract

**Background:**

Emergency departments are often chaotic environments where delays can significantly impact patient care. Key items are stored in supply carts in or near patient rooms to promote efficiency and enable nurses to spend more time assisting patients. However, disorganization, lack of standardization, and lack of stocking can cause significant delays and negatively impact the quality of care.

**Objective:**

This study utilized human-centered and participatory design to improve the workflow for supply acquisition in an emergency department.

**Methods:**

Using a mixed methods, participatory design approach following the double diamond framework, the team worked with nursing staff and physicians in an urban emergency department to understand the root causes of frustrations with the current supply carts. Qualitative findings about bedside nursing workflows were integrated with quantitative observations of inventory and supply usage to drive a rapid-cycle prototyping process to optimize supply management in the bedside cart.

**Results:**

A lack of clinical staffing exacerbates preexisting challenges with restocking the medical supplies in the bedside carts. This problem is compounded by the misallocation of supplies, with high-frequency items underrepresented and low-frequency items overrepresented in the bedside carts. This leads to wastage of the seldom-used supplies and a lack of access to the most used supplies. The reorganization of the cart through co-design with nursing staff sped up supply acquisition by approximately 20% overall, tripled the availability of the most important supplies, and reduced the need for restocking from once per shift to once per 3 shifts, thus producing tangible improvements even within institutional limitations.

**Conclusions:**

A participatory design process, using human factors principles in tandem with extensive input from end users, enables improvements to stocking. Implications for practice include (1) lack of easy access to appropriate supplies negatively impacts patient care and contributes to nurse burnout and frustration, (2) human factors engineering can improve access to patient care supplies through redesigning the layout of hospital supply carts to better align with workflows, and (3) co-design with frequent collaboration from stakeholders and end users ensures that solutions address the issues that matter most in a sustainable way.

## Introduction

In a busy emergency department (ED), every second counts. Medical professionals require access to key supplies and medication in an efficient and timely manner [1,2]. These key items are often stored in supply carts in or near patient rooms in order to promote efficiency [3-6]. However, disorganization and lack of standardization can delay patient care and negatively impact outcomes [1-3,7-9]. The lack of supplies is also a frequent cause of operational failures. Studies have shown that access to supplies is a leading factor in “time wasted” in an emergency setting [3,9,10], and a lack of familiarity with a cart system has been identified as one of the most common factors adversely affecting the quality of care in a high-stakes medical setting [11].

Human factors engineering has emerged as a dominant method to address challenges in stocking [1,2,4,7,8,12-15]. For instance, one study found that reorganizing and standardizing the contents of a resuscitation cart in a children’s hospital according to the 5S Lean design method reduced the time needed to acquire supplies by 46% [7]. Similarly, the reorganization of a hospital medication cart based on usability testing and simulation reduced the number of wasteful actions taken while searching for the proper supply [12].

While groups vary in the methods they used, one thing remains true in each case: the first step requires understanding who is most affected by the challenges caused by the existing system.

The burden of a disorganized supply system often falls heavily on nursing staff, who are already struggling with staffing shortages and heavy workloads [16,17]. Nurses report frequently having to leave rooms to search for supplies, increasing job frustration, delaying patient care, and reducing the time available to spend with patients [9,18]. One study found that more than one-third of nurses’ time is spent looking for equipment, with an annual estimated cost of US $1 million in lost time per year [10].

Nurse feedback and input are therefore a crucial component of any redesign [14,15], both to optimize any new system based on nurses’ lived experience [17] and to promote approval and uptake of a new system [1,19,20]. Nurse feedback and participation also give nurses a voice in their workplace, increasing satisfaction, improving work performance, and helping to provide an environment for safe, quality patient care [14,21].

Here, we discuss the use of a participatory human factors approach to redesign the bedside cart supply in the ED of a low-resourced urban community hospital. The aim of this mixed methods co-design intervention was to understand the challenges associated with restocking and access to patient care supplies and implement a strategy to improve reliable and efficient access to supplies for nurses in an ED. Our novel approach, which combined the use of the double diamond design framework with a participatory design, allowed us to reduce the time required to access supplies by reorganizing the carts to hold more of the most crucial supplies and minimizing wasted space.

## Methods

### Overview

In this paper, we report on the use of a data-driven and participatory design approach to address the supply shortage in ED bedside carts. Our approach followed the double diamond design framework [22]. The double diamond design framework is a human-centered framework that breaks a design into 2 main phases, each composed of a divergent step of discovery and ideation, followed by a convergent step distilling findings into a concise and workable next step. In addition, we relied heavily on principles of co-design [23], working closely with stakeholders at every step of the process to ensure any solutions fit within the constraints of the setting and appropriately addressed their concerns.

Following this framework, the design process was broken into 3 main phases. In the first phase of the double diamond, the team immersed itself in the clinical environment and engaged with stakeholders to gather information. Based on these observations, they then performed a root cause analysis to discover and define the true problems affecting supply accessibility. Finally, they underwent a co-design process with hospital staff to develop and deliver a new bedside cart prototype that successfully addressed the issue. The results are organized according to the SQUIRE (Standards for Quality Improvement Reporting Excellence) reporting guidelines for quality improvement in health care [24].

### Study Background and Location

This study was conducted in the Emergency Department of the Hospital of the University of Pennsylvania’s campus at Cedar Avenue (HUP-Cedar), a low-resourced urban ED in eastern Pennsylvania. The ED serves more than 48,000 patients every year and has inpatient medical and psychiatric services, with very little specialty coverage. Children, patients who are critically injured, or patients with trauma are generally sent to other hospitals in the region, and the admission rate for patients is 10% to 13%. The department is split into a 23-bed high acuity area staffed with a 1:4 nursing to patient ratio and a 10-bed low acuity area staffed with 1 to 2 nurses.

**Figure 1.**
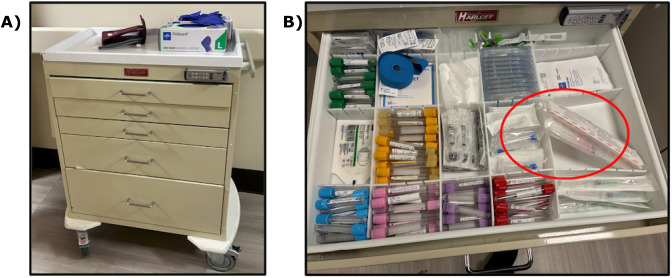
Original cart system. (A) Lockable 5-drawer Harloff ‘‘bedside” cart positioned in the hallway of the emergency department. (B) Original layout and dividers in drawer 1 (topmost drawer) of the bedside carts. Note that needles (indicated by the red circle) are stored at a diagonal because the allocated space is too short.

The ED utilizes a lockable 5-drawer Harloff ‘‘bedside” cart positioned in the hallway outside each patient room. Drawers have varied heights; the top 3 are each approximately 2.5″ tall, the fourth drawer is 5.5″ tall, and the bottom drawer is 8.5″ tall. Supplies are separated by thin, ⅛″ acrylic dividers in compartments that often fail to accommodate longer items such as needles and syringes (Figure 1). In addition, heavy items such as intravenous (IV) bags have deformed many of the carts over the years. Carts are often understocked given limitations in staff availability, causing nurses to search 3 or 4 carts to find necessary supplies. In addition, the underutilized supplies may linger in the cart for years well past their expiration dates. The research team was embedded within the ED from January 2025 to May 2025; the full process can be seen in Figure S1 in Multimedia Appendix 1. The team members included multiple engineers with specialties in mechanical design, product design, data science, human factors, and systems engineering and 2 emergency medicine physicians. The project also included a nurse who provided significant input and helped the embedded team interface with other members of the nursing staff.

### Clinical Immersion and Key Stakeholder Interviews

The team began by observing users interacting with the bedside cart and interviewing key stakeholders (Multimedia Appendix 2) to understand the key challenges and concerns that they saw with the current carts, and to understand the supplies they needed the most. Interviews were semistructured [25] with a predetermined list of questions, but conversations varied according to participant interests. Interviews were conducted with multiple team members present; 1 person led the conversation, while others took detailed notes to ensure no information was missed. After 60 hours of observation, and after the insights from observation and interviewing had reached saturation [26,27], the team used a thematic analysis [28] to identify the key unmet needs (Table 1). These needs were subsequently validated through conversations with nursing staff.

**Table 1.** Sample thematic analysis of user statements.

Insight	Example statements
Unreliable or unavailable supply puts patient lives at risk.	*[When I try to use the carts,] the things I need to save my patient’s life aren’t there.* *I’d rather save patient lives than have a clean cart. I can clean the cart later, but I can’t bring them back.* *I can’t leave a seizing patient to go look for oxygen masks because my cart didn’t have any.*
Stocking is time-consuming and irritating, and there is often no one to do it.	*The techs are pulled to do other tasks like 1-on-1, and no one has time to restock the carts.* *If we had safer ratios, I’d be more willing to stock the carts.* *I just want my carts stocked. The rest, I can work with.* *Anything to make things a little easier or streamlined, especially with refilling the carts, would be huge.*
Nurses do not trust the carts to have the supplies they need and often use workarounds.	*If cart supplies are low, I’ll get a basin of the most used things and put them on a cart so I know where to find them.* I run from cart to cart all day, then say ‘F** it, I’m just going to the stockroom’. *I get things from the Clean Supply rather than the carts so I don’t waste my time looking.*
There is not a perfect consensus over what should be in the carts.	*I’ve been going from drawer to drawer looking for socks for my patient.* *After the emergency, that’s when we can put on socks.*
Cart contents do not match what nurses actually need.	*A lot of things in the cart are rarely used - they’re a waste of space.* *I really want a space for the ultrasound needles.* *When there are things in the cart that aren’t used, they expire and we have to throw them out.*

### Co-Design With Key Stakeholders and Initial Solution Selection

Focused solutions to the supply problem were addressed in a series of co-design sprints to determine how to reform the supply distribution system. A multidisciplinary group of stakeholders (including the research team, additional physicians, and nurses) underwent a series of design thinking brainstorming exercises to generate over 40 potential solutions. From there, ideas were screened for viability (whether the solution addressed the core problem), desirability (whether the stakeholders would like and use the solution), and feasibility (whether the solution would be possible given other constraints). From the session, the team identified 6 key domains—accessibility, legality, reliability, ease of restocking, capacity, and feasibility—required for a successful solution based on the unmet needs. Subsequently, screening and scoring matrices were implemented to identify the top solution [29]. Although the original solution selected was to pull out the key items used most often by nurses and put them in their own designated carts, obtaining additional carts would have become a challenge. The team sought to determine whether a similar result could be obtained merely by reallocating the proportions of various items in the existing carts to align with usage and need.

### Quantitative Analysis of Current Inventory System and Item Usage

An inventory of 6 different carts throughout the ED was obtained to understand the current supply state. Maximum capacity was calculated as the highest value from each of the carts. During the observation period, the team also collected data on the supplies used to care for 17 different patients with different chief complaints (16 h of observation). This allowed the team to see supplies being used in real time and observe nursing micromovements as well as get a sense of overall inventory throughout the department.

From there, the team ran a k-means clustering analysis on the supply usage data to identify if there were any clusters of supplies that were frequently used together. While preliminary data were too limited to generate many solid clusters, the initial results using k-means with 2 clusters revealed a small cluster of high-use items and a much longer list of less frequent items. These high-use items were then compared with reports given by nurses (Multimedia Appendix 3).

### Rapid Prototyping of New Drawer Organization System

**Figure 2.**
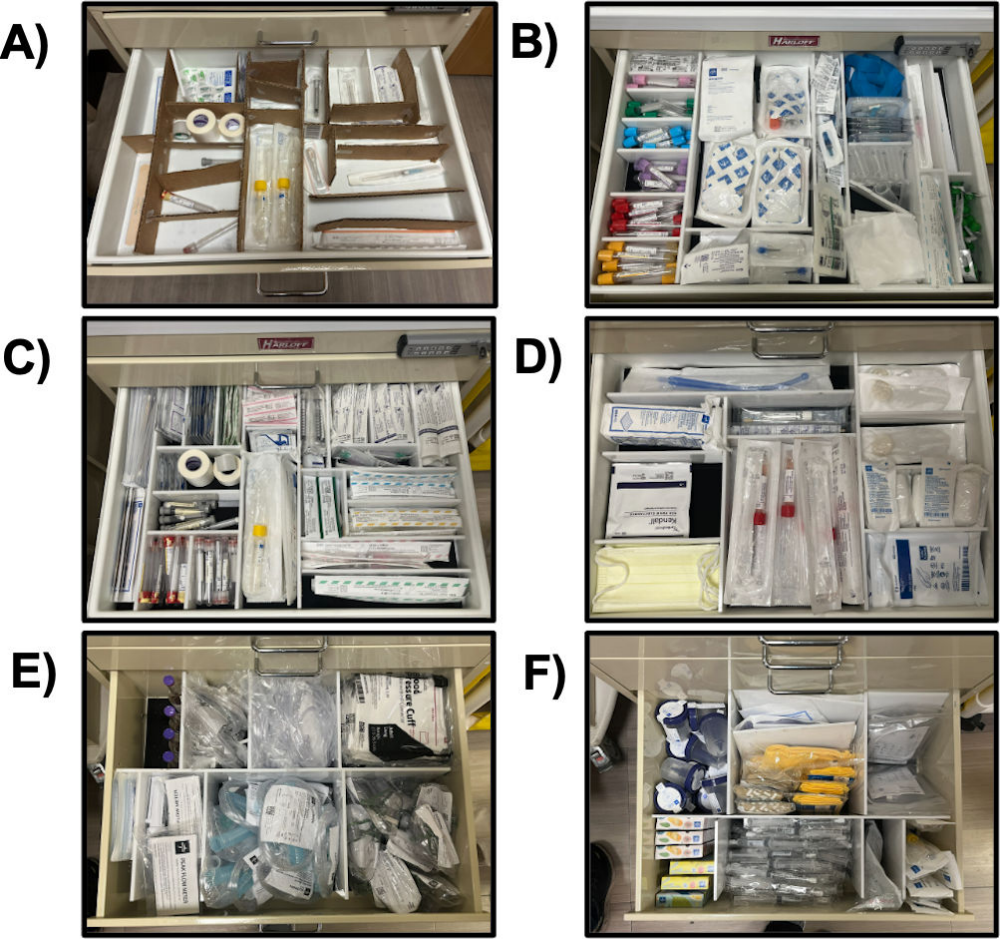
Prototype to finalized product. (A) Cardboard prototype used in an interactive design process with nurses (drawer 1); (B) finalized, fully stocked prototype for drawer 1; (C) drawer 2; (D) drawer 3; (E) drawer 4; and (F) drawer 5.

Through conversation with nursing staff, the team obtained (1) a list of all of the supplies nurses wanted in the supply carts, (2) suggestions for changes in quantity for items already present, and (3) requests for layout changes to improve workflow. The team compared the wish lists to the inventory lists and eliminated unnecessary items, then integrated the qualitative suggestions with their previous quantitative results to create prototypes for drawer dividers to better allocate cart space. This was done in a “jigsaw” fashion [8]: the team built early prototypes from cardboard and tape using real supplies as a guide, then asked the nurses to move around the dividers as needed (Figure 2A). The team also asked nurses to model the movements needed to acquire items so that the new compartments had an ergonomic flow.

Prototypes were designed according to the following principles, all based in human factors engineering: (1) items that were used together should be located in the same drawer and in close proximity, (2) higher-frequency items should be on higher shelves to optimize body posture, (3) compartments should conform to the size and shape of the item, (4) items should fit enough supply for at least 1 full shift for a nurse, and (5) designs should minimize exterior changes to the cart to minimize relearning.

Once nurses provided feedback on the cardboard prototypes, the team incorporated those changes into new prototypes (Figure 2B-E). Team members created computer-assisted design drawings using OnShape [30] for each of the drawer bases and inserts based on the final cardboard models, then laser-cut prototypes made from ¼″ acrylic using a laser cutter.

### Simulation and Evaluation of Design

To ensure that the dividers were an improvement over the original models, the team ran a variety of simulations with the help of nursing staff. The team created four scenarios that would require different combinations of supplies: (1) a patient ready for discharge, (2) a patient with fever and a recurrent headache, (3) a patient with chest and abdominal pain, and (4) a patient with multiple constitutional complaints. The number of scenarios was based on nurses’ estimates of how many patients they would see in each room throughout the duration of their 12-hour shift; scenarios were intentionally left vague.

The team fully stocked both an existing cart and the prototype and asked on-shift nurses to collect the necessary supplies for each of the patient scenarios. Items removed from the cart were not replaced but instead set aside; nurses were told that if an item was not present in the cart when they needed it, they should go about their normal routine, whatever that was. This often involved searching another cart, or going to the clean supply, substituting a different item or collection of items, or foregoing that item altogether. Nurses were quasi-randomly assigned, in equal numbers, to start the simulation with either the prototype cart or the current standard to grab items from either the old or new carts first so that the time required to figure out what items to select did not bias the overall time. When all 4 scenarios were conducted with 1 cart, they would then run the same scenarios with the other cart.

Carts were not restocked between sets of simulations (“shifts”). This was to mimic real-time usage, where stockers often would not be able to resupply carts until multiple shifts had elapsed. After 3 shifts had elapsed without restocking, the carts were fully restocked, and the simulations were run again with different nurses.

### Ethical Considerations

The University of Pennsylvania’s Institutional Review Board (IRB) waived the need for ethics approval and participant consent for the collection, analysis, and publication of the anonymized data for this quality improvement initiative. Informed consent was obtained verbally before participation. All data have been anonymized. No identifying information about the nurses was collected, and the team was not assessing their responses for clinical accuracy. Nurses were not compensated for their participation.

## Results

### Clinical Immersion and Key Stakeholder Interviews

The team spoke with a wide range of stakeholders, hoping to engage with people throughout the entire supply workflow. This included nurses, doctors, and physician assistants who used the carts; technicians who stocked the carts; and even the supply manager who oversaw stocking and ensured that the department remained in compliance with hospital policy and external regulations. The full chart of interviewees can be found in Multimedia Appendix 2.

 During the observation period, the team was told that the bedside carts were fully stocked by emergency technicians and were positioned outside patient rooms so that nurses could efficiently provide patient care. In practice, however, carts were constantly understocked, and nurses frequently visited multiple carts searching for supplies. As a result of this, some nurses no longer relied on the carts and instead went to the central supply closet every time they needed something or carried a personal bucket of supplies they thought they might need, contrary to hospital policy.

After roughly 60 hours of observations and thematic analysis of statements obtained during the interviews (Table 1), it became clear that the current setup did not meet the supply and demand for current nursing staff. For example, each cart could hold only 2 IV start kits, while nurses reported using at least 1 per patient. In addition, the cart could only hold up to three 1-L bags of IV fluids, when a nurse went through an average of 5 to 10 bags per shift across all of their rooms. As a result, these key supplies were constantly in short supply and frequently depleted.

Second, while technicians were responsible for restocking these carts, they lacked sufficient time to do so amid widespread staffing shortages and a host of other, often more critical responsibilities such as sitting with patients who required continuous monitoring or helping to transport patients. Nurses could assist with restocking when time permitted, but ED shifts were often busy, and this was usually impossible. Carts often remained unstocked for several shifts, often for days on end.

In addition, while there was a photographic guide on top of the cart to assist with stocking, there was a lack of consistency in the items inside the cart. Over time, nurses had begun to stock the carts with their preferred items and formats. The clean supply room was aptly labeled with items in their respective categories—IV, wound care, comfort, respiratory, fluids, and so forth—but this system did not correspond to the organization in drawers.

Finally, the observations and interviews demonstrated the importance of buy-in for any potential solution. Nurses expressed concern that any additional work from a proposed solution would fall upon their already-overloaded shoulders and stated outright that there were certain proposals that they would simply not implement.

### Co-Design With Key Stakeholders and Initial Solution Selection

The interdisciplinary team held a design sprint workshop with several nurses to generate over 40 potential solutions. Solutions ranged from developing new technologies and processes to detect when a cart was empty, to hiring a new person with the sole job of restocking carts, to altering the workflows of staff members in other departments to help assist with restocking, to even assigning each nurse their own cart to be responsible for and stock as they wished. However, solutions were limited by resources and by staffing shortages and workload. The hospital was unable to allocate money to hire a new person or replace the existing carts, and the team did not identify anyone in the environment with the extra time and ability to add restocking to their workflow. In addition, policy restrictions meant that each cart needed to be standardized. Additionally, nurses and doctors rejected any solution that involved making restocking their responsibility entirely. Given these limitations, the most feasible solution involved a reorganization of the cart system, utilizing the existing space but reconfiguring it to increase capacity for high-use items.

### Quantitative Analysis of Current Inventory System and Item Usage

Before making any attempts to redesign the cart capacity, the team needed to understand both the original layout and typical patterns of item usage. The team thus developed an inventory to track supply usage for nurses across a total of 16 hours to capture the relative frequency of supply usage, as well as the types of supplies often used in conjunction with each other. From this information, and through consultation with nursing staff, the team was able to isolate 3 key clusters of highly used supplies: IV, diagnostics, and respiratory supplies. The contents of each of these clusters can be found in Multimedia Appendix 3. They also obtained an approximate estimate of the amount of each of these supplies needed per day. IV start kits were the most highly used item, as they were used with nearly every patient. Along with the kits, nurses also used flushes, heplocks, and vacutainers.

### Rapid Prototyping of New Drawer Organization System

**Figure 3.**
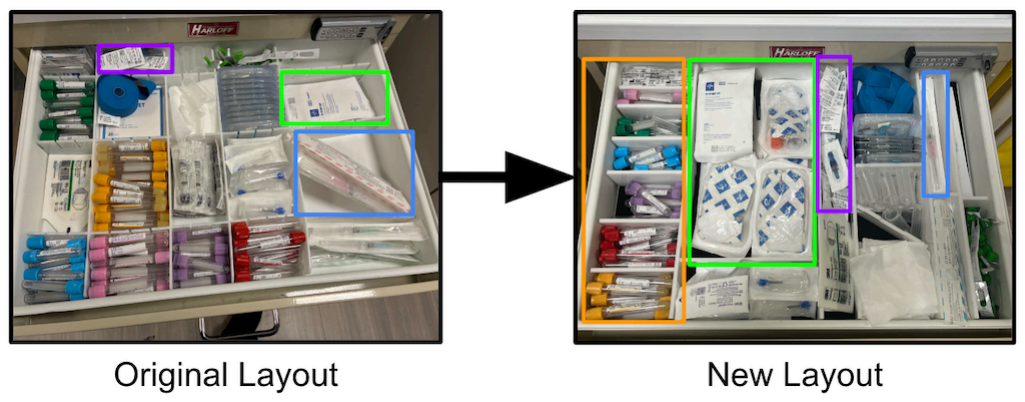
Drawer 1 original versus new layout. Colored boxes highlight key changes that were made. Blue: Needles are no longer stored at a diagonal with no decrease in capacity. Purple: Hep-lock capacity increased by 75%. Green: Intravenous start kit capacity increased by 200%. Orange: Storing all tubes in 1 column makes for a more ergonomic workflow; capacity decreased to avoid wastage.

**Figure 4.**
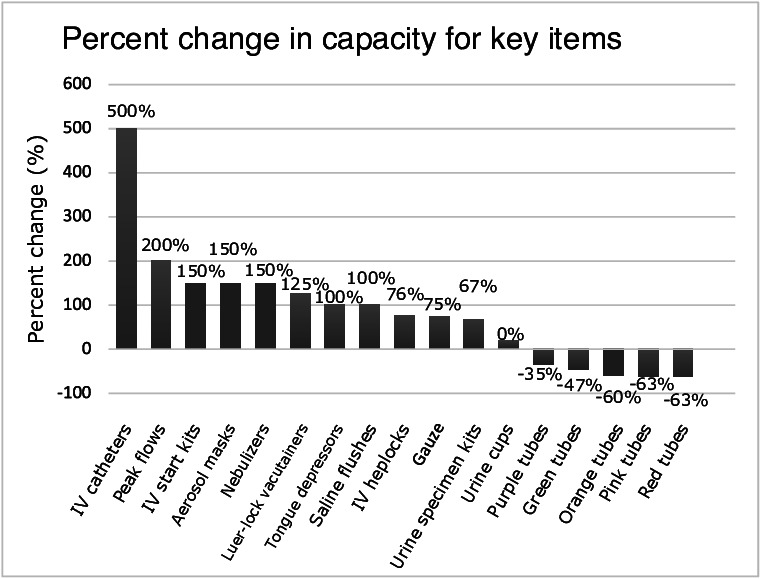
Cart redesign corrects supply allocation. After redesign, critical items like those used with intravenous (IV) or for urine collection and testing increased in capacity, while overrepresented test tubes decreased in capacity.

After undergoing the rapid prototyping process described above and shown in Figure 2, the new design provided a marked improvement over the old model. The layouts for the old versus original designs can be seen in Figure 3, and the difference in capacity between the 2 designs can be seen in Figure 4 (key supplies only; full list in Multimedia Appendix 4). Specifically, for the IV kits, the new carts comfortably held 9 kits, while previous models held only 2 or 3—a 200% increase for the most highly used supply. Co-occurring supplies, such as the flushes (100% increase), vacutainers (125% increase), and heplocks (75% increase), similarly increased in capacity. Given the total number of carts on the floor, this meant that the number of IV kits stored in the carts jumped from approximately 100 to approximately 300—enough to make it through multiple shifts without restocking. The new cart design similarly doubled the size of spaces allocated for key respiratory supplies such as peak flow meters (200% increase), nebulizers (150% increase), and aerosol masks (150% increase).

In many cases, this was achieved by removing low-frequency items that were rarely used and could be more effectively stored in the central supply closet. In other cases, it was achieved by ensuring each compartment fit the size of the tool it was meant to contain, eliminating wasted space and reducing the chances of needles being damaged or bent by attempting to force them into the cart. The new design also allowed space for the addition of other supplies, such as a speculum, that were highly sought after by physicians and nurses and could not fit in the cart previously. The design met with approval from nursing and administrative staff at all stages of the process.

In addition, the finalized model was both sturdier and less prone to breakage than the original model. This was the result of several iterations: the first draft, which used an acrylic solvent, proved unstable and snapped upon construction, so the team reconfigured the designs to use a press-fit layout instead. With this approach, drawers were easy to assemble and sturdy enough to withstand constant use.

### Simulation and Evaluation of Design

Once the prototypes were completed, they were tested for practical usage. After completing the simulation described above, the novel carts outperformed their older counterparts. On average, nurses were able to acquire needed supplies from the new cart nearly 6 seconds faster (21% faster) than they were able to get supplies from the old cart (2-tailed paired *t* test, time to acquire item from old cart=38.2 seconds; time to acquire item from new cart=32.4 seconds; degrees of freedom=23*; P*=.097). The lack of statistical significance was likely a limitation of low sample size; in addition, this simulation likely overestimated the time required to get samples from the newer cart because nurses were unfamiliar with the new configuration.

**Figure 5.**
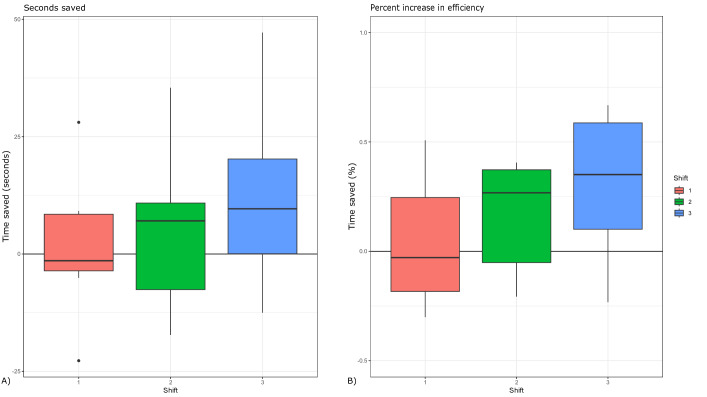
Time savings as a function of new cart. (A) The new cart saved an average of 5.8 seconds per simulated patient, and the time saved increased as the cart went longer without being restocked. (B) As the cart went longer without restocking, the percentage of time saved while accessing needed supplies increased, from about even when both were fully stocked, to 40% after 2 shifts. Boxplots show distribution of data (n=8 per shift). Sample size was too low to assess statistical significance.

In addition, the time saved only increased as the carts went longer intervals without needing to be restocked. During the first shift, the new cart only saved about 1.5 seconds on average. During the second shift, the new cart saved an average of 4.7 seconds. On the third shift, the cart saved an average of 11.3 seconds (Figure 5). This time is a direct reflection of an improvement in capacity. While the old cart ran out of IV start kits by the third patient, forcing nurses to search for kits in nearby carts for every subsequent patient, the new cart contained all of the needed supplies for every patient. On average, a trip from a cart to the clean supply room (the preferred solution for nurses who did not trust that supply carts contain the proper equipment) took 1 minute; these trips were entirely eliminated by the use of the new prototype.

Furthermore, the team noticed that the number of supplies taken from the new cart was much higher than those taken from the old cart. Specifically, there were several items that nurses were not willing to go to another cart for, such as speculums or certain test kits, but were happy to grab if they were present. This implied that having carts stocked with these supplies meant that nurses would actually use them, providing more timely patient care.

### Plans for and Barriers to Full Implementation

Due to resource constraints, the new drawers are not fully implemented throughout the department. Full implementation would require a large up-front cost: due to the reorganization of supplies, some supplies were moved to different drawers. Because hospital policy requires that all carts contain the same organization, it is not possible to gradually phase in new carts over an extended period. In addition, because of this reorganization, it is not possible to replace the cart drawers for all carts, 1 drawer at a time, since incomplete adoption would mean that some items would no longer be included. This is certainly a limitation of the team’s design process—had considerations about gradual or modular adoption been considered earlier in the process, this would not have been an issue. However, the team is working to find the lowest-cost way to implement these new designs.

They are also working to mitigate unintended consequences arising from adjustments to the new cart system. Specifically, to improve the ease of adjustment, they have created a pamphlet showing an updated schematic of the cart with pictures of the contents of each full cart. A written list of the cart’s contents and approximate capacity will also be provided to keep at each nursing station for training purposes and reference. They will label carts with the contents of each drawer (ie, drawer 1 will be labeled with IV supplies; drawer 2 will be labeled with wound care, diagnostics, and extra needles; drawer 3 will be labeled with miscellaneous small supplies; drawer 4 will be labeled with respiratory supplies; and drawer 5 will be labeled with flushes, urine cups, catheters, tubing), and the bottom of each section of the drawer will have a photo of the supply that belongs inside it. These improvements will facilitate stocking, assist with the transition, and make it easier to onboard new nurses. In addition, it will decrease the likelihood of drift over time, ensuring that staff use the partitions as directed.

## Discussion

### Principal Findings

This participatory design process highlighted the importance of co-design for improving workflow and supply management in an ED. By observing, interviewing, and directly working with the people who use the system the most, the team was better able to understand the key challenges that influenced the effectiveness and constrained the solution space. The final prototype increased the capacity for key items by up to 200%, enabling cart capacity to last 36 hours (3 shifts) rather than requiring restocking within the first 12-hour shift. In addition, similar to other studies [15,31], the reduction in response time finding supplies did not reach statistical significance, but trended toward a statistical result. This is in spite of the fact that the new design meant participants spent more time looking for objects that had been moved, a metric that would improve as users became more familiar with the new system. Notably, the improvement here is likely due to the new design reducing the number of trips to the clean supply room or searching for supplies from other carts, rather than from the more logical organization of the cart itself.

Overall, the findings of improved supply accessibility are in line with reports from other human factors–based redesigns of supply carts in other departments of the hospital [1,2,4,7,12-14]. While the constraints of this setting are not entirely analogous to other settings—here, the challenge was less about disorganization and more about a limited availability of stockers—they nevertheless suggest that cart organization, tailored to the needs of the staff and built with human factors principles in mind, plays a critical role in departmental workflows.

Even when the carts were fully stocked, nurses noted that the items that were present were there in the wrong quantities. Low-frequency items were present in large quantities, while there were hardly any of the most commonly used supplies. In fact, the supply manager told us that she had gone through the carts and had to toss hundreds of dollars’ worth of unused supplies that had expired, an enormous waste of money and space for a department that had little to spare of either. This finding of overstocked items leading to wastage has been found in other sites as well [3,32] and suggests that periodic evaluation to ensure that carts can contain appropriate quantities of each supply is necessary. Our discovery of clusters of items frequently used together also matches the findings reported elsewhere [33].

In addition, the success of the quality improvement demonstrated the importance of participatory design, particularly the frequent consultation with stakeholders during the ideation and prototyping process, ensuring that the team’s proposed solution was both effective and acceptable. Stakeholders were glad to be able to address a problem that mattered a great deal to them and helped to ensure that the new designs addressed their particular concerns about the old model. Co-design processes and design sprints are widely used in applied health research and are critical for ensuring that solutions focus on outcomes that are most important to end users [23,34,35].

Tailoring the cart’s contents to the user workflow, and specifically minimizing the number of drawers that needed to be opened, improved user experiences. Although the cart’s organization changed slightly, and users were less familiar with it, they still found it easier to use and preferred it over the standard cart. This is similar to prior findings that nurses preferred to use a cart that only required them to open 1 drawer per patient rather than a cart where everything was organized by tool but required multiple accesses [13].

In addition, nurses discussed how eager they were to be able to participate in the process. While this has been a persistent problem, engaging nurses in the redesign process gave them a voice, helping them feel like change was possible. This matches with previous research that found that nurse involvement in the co-design process improves nurse satisfaction [14].

The prototype created by this team is an example of how participatory design can create substantial improvement in a nursing workspace. The team was able to identify the key items nurses needed most and provide enough of those items to last multiple shifts without restocking, substantially improving workflow. The organization was designed according to nurses’ priorities, focusing on ergonomics and efficiency using the same principles found in other studies [14,15]. While the solution could not solve the root causes of the issue—low funding and staffing shortages—it still made a meaningful, actionable contribution within the preexisting constraints by reducing the frequency of needed restocking. Once fully implemented, it is expected to save nurses a substantial amount of time per shift looking for items, reducing frustration and increasing the amount of time available for providing quality patient care. In addition, the durability of the acrylic, the work done to document and improve the ease of replicability of the system, and the buy-in from stakeholders all help to cement its durability and reduce the drift in restocking practices.

While the final design of the drawers in this project is customized to the specific hospital, the co-design process used demonstrates how stakeholder contribution and co-design can enable teams to make a meaningful impact in a low-resourced setting, working around structural barriers to improve nurse workflow and patient care.

### Limitations

This project, while useful, was unable to address the root cause of the problem of supply shortages: the lack of a dedicated person who could simply restock all of the carts on a reliable basis. Despite this challenge, the implemented solution managed to ameliorate these effects by decreasing the time needed to restock and increasing the time the cart could go without restocking.

The project also encountered challenges regarding implementation—specifically, that because some items were moved to different drawers, and institutional policy required that all carts be organized the same way, implementation would need to occur all at once rather than being phased in gradually, requiring a larger up-front cost.

In addition, the data collection for the simulations was limited due to team availability and the availability of nursing staff to assist during their own busy shifts. The simulations also relied on convenience sampling: working with whatever staff were available at the time. Staff received the same prompts for both carts in the simulation; it is possible that thinking through the supplies beforehand made them faster at accessing the supplies the second time. We attempted to mitigate this response by randomizing which cart the team used first. Variable levels of experience with both the old and revised carts may also have led to bias in how quickly nurses found supplies in each cart [15]. Despite the lack of significance in time saved, the team was able to validate the use of the intervention through the increase in capacity for key supplies and through positive qualitative feedback from nursing staff indicating that, based on their past experiences, the cart would make a substantial improvement.

Finally, the designs for the cart interiors created during this project were tailor-made for this specific department and the cart system already in place, and would not necessarily generalize to other EDs. However, the processes used to design and validate the solution are readily transferable, and the designs could be tweaked to fit the unique contexts of other departments.

### Conclusion

The co-design process used in this study demonstrates the importance of stakeholder engagement and contribution to quality improvement and the use of the double diamond approach in a health care setting. Stakeholder input was crucial for navigating around institutional barriers and ensuring that any proposed solutions would be accepted and implemented, rather than ignored. Indeed, the buy-in generated from participatory design ensured that the changes made would be sustainable. While the team’s designs were specific to the ED’s context, the process and insights can be applied to any other type of workplace improvement process. Specifically, we recommend engaging with stakeholders at every step of the way, particularly during the rapid prototyping phase. However, we suggest that future teams consider the ease of implementation, especially gradual implementation for budgetary reasons, while evaluating their designs.

## Supplementary material

10.2196/80861 Multimedia Appendix 1 Timeline of steps for cart intervention project.

10.2196/80861 Multimedia Appendix 2 List of interviewees for primary stakeholder interviews.

10.2196/80861 Multimedia Appendix 3 High-use items identified by clustering algorithm and nurse report.

10.2196/80861 Multimedia Appendix 4 Comparison of the number of items in the current nursing carts to the modified amounts in the prototype (complete inventory).

## Abbreviations

ED: emergency department
IV: intravenous
SQUIRE: Standards for Quality Improvement Reporting Excellence
