# Supplementary figures and images for: Redesign of Bedside Supply Carts to Improve Emergency Department Workflows: Mixed Methods Participatory Design
Source: JMIR Hum Factors. 2026 Jan 28;13:e80861. doi: 10.2196/80861 (PMC12850040; doi:10.2196/80861)

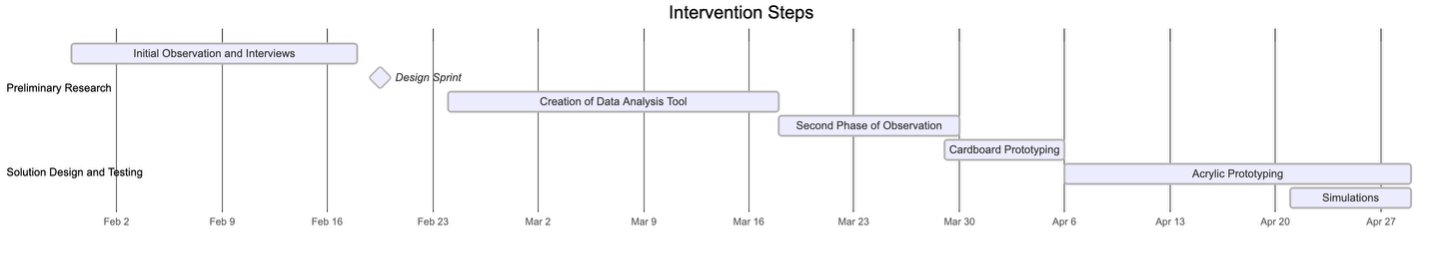

Supplement: Multimedia Appendix 1 [file humanfactors-v13-e80861-s001.png]
