# Supplementary material for: Redesign of Bedside Supply Carts to Improve Emergency Department Workflows: Mixed Methods Participatory Design
Source: JMIR Hum Factors. 2026 Jan 28;13:e80861. doi: 10.2196/80861 (PMC12850040; doi:10.2196/80861)
Supplement: Multimedia Appendix 2 [file humanfactors-v13-e80861-s002.docx]

**Table S1:** List of Interviewees for Primary Stakeholder Interviews

| **Role** | **Count** |
| --- | --- |
| Nurse | 8 |
| Technician | 2 |
| Resident | 2 |
| Supply Manager | 1 |
| Physician Assistant | 1 |
| Attending Physician | 1 |
