# Supplementary material for: Redesign of Bedside Supply Carts to Improve Emergency Department Workflows: Mixed Methods Participatory Design
Source: JMIR Hum Factors. 2026 Jan 28;13:e80861. doi: 10.2196/80861 (PMC12850040; doi:10.2196/80861)
Supplement: Multimedia Appendix 3 [file humanfactors-v13-e80861-s003.docx]

**Table S1:** High-Use Items Identified by Clustering Algorithm and Nurse Report

| **Clustering Algorithm** | **Nurse Report** | **Major Spatial Clusters** |
| --- | --- | --- |
| Most highly used items:   - IV start kit - Luer-lock vacutainer - Heplock - 18 gauge IV needle - Flushes - Green test tube - Blue test tube - Orange test tube - Pink test tube - Purple test tube - Red test tube - 4x4 cotton gauze - Lube - 3 ml syringe | Top 5:   - IV start kit - Luer-lock vacutainer - Heplock - 18 gauge IV needle - 20 gauge IV needle | IV and Related (Drawer 1):   - IV start kit - Luer-lock vacutainer - Heplock - 18 gauge IV needle - 20 gauge IV needle - IV test tubes (green, blue, orange, pink, purple, red) - 4x4 cotton gauze |
|  | Additional concerns:   - Flushes - Green test tube - Blue test tube - Orange test tube - Pink test tube - Purple test tube - Red test tube - Oxygen mask - Nebulizer - Nasal cannula - Aerosol mask - Grey test tube - Red and yellow test tube - Clear test tube   Urine cups | Diagnostics (Drawer 2):   - Test tubes (red/yellow, grey, clear) - Urine test |
|  |  | Respiratory (Drawer 4):   - Oxygen mask - Nebulizer - Nasal cannula - Aerosol mask |
|  |  | Bulky Items (Drawer 5):   - Urine Cups - Flushes |
