# Supplementary material for: Redesign of Bedside Supply Carts to Improve Emergency Department Workflows: Mixed Methods Participatory Design
Source: JMIR Hum Factors. 2026 Jan 28;13:e80861. doi: 10.2196/80861 (PMC12850040; doi:10.2196/80861)
Supplement: Multimedia Appendix 4 [file humanfactors-v13-e80861-s004.docx]

**Table S1:**  Comparison of the number of items in the current nursing carts to the modified amounts in the prototype (complete inventory).

|  | **Item** | **Request** | **Current** | **Prototype** | **Difference** |
| --- | --- | --- | --- | --- | --- |
| **Drawer 1** | Alcohol prep pad |  | 55 | 35 | -20 |
|  | Blood collection tube  (Green top) | Less | 30 | 16 | -14 |
|  | Tegaderm |  | 45 | 32 | -13 |
|  | Blue test tube | Less | 25 | 12 | -13 |
|  | IV Heplock | More | 17 | 30 | 13 |
|  | Tourniquet |  | 2 | 2 | 0 |
|  | Blood collection tube  (Orange top) | Less | 40 | 16 | -24 |
|  | Blood collection tube  (Pink top) | Less | 24 | 9 | -15 |
|  | Clear empty tube | Less | 24 | 18 | -6 |
|  | 4x4 cotton gauze |  | 20 | 35 | 15 |
|  | VBG Aspirator | Less | 12 | 6 | -6 |
|  | blood collection tube  (Purple top) | Less | 20 | 13 | -7 |
|  | IV disinfecting cap | Less | 50 | 33 | -17 |
|  | Push Button Blood Collection Set | Less | 20 | 6 | -14 |
|  | Luer-lock Vacutainer | More | 8 | 18 | 10 |
|  | Blood collection tube  (Red top) | Less | 30 | 11 | -19 |
|  | IV start kit | More | 4 | 10 | 6 |
|  | 20-gauge needles |  | 9 | 10 | 1 |
|  | 18-gauge needles |  | 8 | 8 | 0 |
|  | 16-gauge needles |  | 1 | 0 | -1 |
| **Drawer 2** | Tape |  | 14 | 5 | -9 |
|  | Tongue depressors |  | 20 | 40 | 20 |
|  | Cotton tip swabs |  | 39 | 40 | 1 |
|  | Urine specimen collection kit | More | 6 | 10 | 4 |
|  | Germicidal Disposable Wipe |  | 25 | 16 | -9 |
|  | Cavillon No Sting Barrier Film |  | 8 | 16 | 8 |
|  | Sterile Lubricating Jelly |  | 21 | 20 | -1 |
|  | Neosporin | Remove | 20 | 0 | -20 |
|  | Bandage - big |  | 5 | 30 | 25 |
|  | Bandage- small |  | 19 | 30 | 11 |
|  | Clear top tube |  | 35 | 21 | -14 |
|  | 1 ml syringe |  | 10 | 8 | -2 |
|  | Insulin syringe |  | 20 | 0 | -20 |
|  | Yellow and red speckled tube | Less | 30 | 21 | -9 |
|  | Grey top urine culture tube | Less | 30 | 18 | -12 |
|  | 14/16 gauge IV Catheters |  | 2 | 12 | 10 |
|  | 18/20 gauge IV Catheters |  | 0 | 12 | 12 |
|  | 22 gauge IV Catheters |  | 15 | 10 | -5 |
|  | 24 gauge IV Catheters |  | 7 | 10 | 3 |
|  | 3 ml syringe |  | 14 | 28 | 14 |
|  | 5 ml syringe |  | 14 | 24 | 10 |
|  | 10 ml syringe |  | 8 | 5 | -3 |
|  | 21-gauge tip |  | 11 | 6 | -5 |
|  | 22-gauge tip |  | 11 | 6 | -5 |
|  | 23-gauge tip |  | 4 | 6 | 2 |
|  | 24-gauge tip |  | 2 | 6 | 4 |
|  | 25-gauge tip |  | 2 | 8 | 6 |
|  | 18-gauge tip |  | 30 | 30 | 0 |
| **Drawer 3** | Conforming stretch gauze |  | 15 | 10 | -5 |
|  | 2x2 gauze |  | 100 | 200 | 100 |
|  | Biohazard lab specimen bags |  | 54 | 60 | 6 |
|  | Telemetry electrodes |  | 10 | 6 | -4 |
|  | Face masks |  | 6 | 20 | 14 |
|  | E-Swab Kit |  | 10 | 10 | 0 |
|  | Covid Swab test |  | 15 | 18 | 3 |
|  | 50 ml syringe | Remove | 3 | 0 | -3 |
|  | 30 ml syringe | Remove | 7 | 0 | -7 |
|  | Yankauer |  | 3 | 4 | 1 |
|  | ChloraPrep applicator |  | 3 | 3 | 0 |
|  | Abdominal pad | Remove | 8 | 0 | -8 |
|  | Pulse Oximeter |  | 8 | 8 | 0 |
| **Drawer 4** | Anaerobic blood culture bottle (Purple Cap/Maroon Ring) |  | 6 | 6 | 0 |
|  | Aerobic blood culture bottle  (Grey Cap/Blue Ring) |  | 6 | 6 | 0 |
|  | Gauze sponge |  | 13 | 20 | 7 |
|  | Drain sponge | Remove | 16 | 0 | -16 |
|  | 7-inch tubing | Remove | 4 | 0 | -4 |
|  | Nasal cannula |  | 10 | 10 | 0 |
|  | Peak flow meter | More | 2 | 6 | 4 |
|  | Aerosol mask | More | 4 | 10 | 6 |
|  | Nebulizer | More | 4 | 10 | 6 |
|  | Non-rebreathing mask | Same | 5 | 4 | -1 |
|  | BP Cuffs | More | 8 | 10 | 2 |
| **Drawer 5** | Purewick Female External Catheter |  | 5 | 2 | -3 |
|  | Urine collection cup | More | 15 | 18 | 3 |
|  | Suction tubing |  | 15 | 5 | -10 |
|  | Male Purewick Catheter |  | 11 | 2 | -9 |
|  | Normal saline IV flushes | More | 30 | 60 | 30 |
|  | Tissue box | More | 1 | 10 | 9 |
|  | Secondary tubing |  | 10 | 8 | -2 |
|  | Primary tubing |  | 5 | 6 | 1 |
|  | Gravity tubing |  | 9 | 5 | -4 |
|  | Socks | More | 3 | 10 | 7 |
|  | Sterile vaginal speculum | Add | 0 | 1 | 1 |
|  | Suction tubing |  | 15 | 8 | -7 |
|  | Lactated Ringer's solution |  | 3 | 0 | -3* |

*****- LR is now placed on top of the cart
